# Correlation of plasma crizotinib trough concentration with adverse events in patients with anaplastic lymphoma kinase positive non-small-cell lung cancer

**DOI:** 10.1186/s40780-014-0008-x

**Published:** 2015-03-02

**Authors:** Yasuko Kurata, Narumi Miyauchi, Manabu Suno, Takahiro Ito, Toshiaki Sendo, Katsuyuki Kiura

**Affiliations:** Department of Pharmacy, Okayama University Hospital, Okayama, 700-8558 Japan; Department of Oncology Pharmaceutical Care & Sciences, Graduate School of Medicine, Dentistry and Pharmaceutical Sciences, Okayama University, Okayama, 700-8530 Japan; Department of Allergy and Respiratory, Okayama University Hospital, Okayama, 700-8558 Japan

**Keywords:** Crizotinib, Therapeutic drug monitoring, Adverse events, LC-MS/MS

## Abstract

**Background:**

Crizotinib, an ATP-competitive receptor tyrosine kinase inhibitor of both anaplastic lymphoma kinase (ALK) and the hepatocyte growth factor receptor, commonly causes several adverse events (AEs). The clinical utility of measuring the plasma concentration of crizotinib in patients with non-small-cell lung cancer (NSCLC) has not been fully elucidated. The aim of this study was to evaluate the variability in the crizotinib trough concentration and its relationship with the occurrence of AEs in NSCLC patients.

**Findings:**

Plasma samples were collected from 9 ALK fusion gene-positive NSCLC Japanese patients at day 14 after the first administration of crizotinib. We assessed crizotinib-induced AEs on days 7, 14, 21, and 28. The crizotinib trough concentration on day 14 ranged from 243.5 to 847.8 ng/mL, and all of the patients achieved stable disease based on assessment of the tumor response on day 28. The cumulative number of AEs on day 28 in the higher trough concentration group was approximately 3-fold greater than that in the lower trough concentration group. AEs of grade 3 or 4 were observed only in patients in the higher trough concentration group.

**Conclusions:**

The occurrence of several AEs may correlate with the increase in the crizotinib trough concentration. Monitoring of the crizotinib trough concentration could predict the risk of development of several AEs and provide guidance for determining the optimal dose of crizotinib.

## Findings

### Background

Several tyrosine kinase inhibitors, including gefitinib, erlotinib, and crizotinib, have demonstrated substantial single-agent activity in the treatment of metastatic non-small-cell lung cancer (NSCLC) [1-3]. Among these drugs, crizotinib is an ATP-competitive receptor tyrosine kinase inhibitor of both anaplastic lymphoma kinase (ALK) and hepatocyte growth factor receptor (c-MET) tyrosine kinases. Crizotinib blocks intracellular signaling pathways and reduces the growth and size of tumors [4-8]. The recommended oral dose of crizotinib is 250 mg twice daily, and a steady-state can be reached within 2 weeks [8,9]. A clinical trial of crizotinib has reported that the maximum drug concentration (Cmax) and area under the concentration-time curve (AUC) of crizotinib in Asian patients were higher than that in non-Asian patients [10]. Although the difference in physical size might influence the plasma concentration of crizotinib, patients undergo treatment with a fixed dose level of crizotinib. Crizotinib causes severe adverse events (AEs), such as leucopenia, neutropenia, elevated alanine aminotransferase (ALT) levels, and QT interval prolongation [10-16]. However, the relationship between the plasma concentration of crizotinib and AEs has not yet been determined in patients. Therefore, measuring crizotinib concentrations in the plasma of patients may be substantially beneficial for predicting AEs and for defining the optimal dose of crizotinib.

In the present study, we quantitated crizotinib in the plasma of patients using a liquid chromatography-tandem mass spectrometry (LC-MS/MS) method and also investigated the relationship between crizotinib trough concentration and crizotinib-induced AEs based on clinical data.

## Methods

### Ethics

This study was designed and implemented following the guidelines dictated in the Declaration of Helsinki. Ethics approval was obtained from the Okayama University Graduate School of Medicine, Dentistry, and Pharmaceutical Sciences Ethics Committee. Patients in this study remained anonymous. Written informed consent was obtained from patients in the prospective study. All patients received medical care and treatment according to the protocols and policies of the hospital without being influenced by this study.

### Patients and plasma samples

Plasma samples were collected from 9 ALK fusion gene-positive NSCLC Japanese patients receiving a daily oral dose of either 400 or 500 mg of crizotinib between August 2012 and July 2013 at Okayama University Hospital. Since crizotinib reaches a steady-state within 2 weeks, we collected plasma samples before the patients had taken crizotinib in the morning at day 14 after the first dose of crizotinib. Venous blood (1.0 mL) was collected in heparinized tubes. The supernatant in the centrifuged tubes were collected and stored at −30°C until analysis.

Patient information, including age, sex, height, weight, performance status (PS), disease stage (based on the TNM classification), and previous chemotherapy for NSCLC were obtained from their medical records. The incidence of AEs was assessed on treatment days 7, 14, 21, and 28. The grades of AEs were assessed using the grading system of Common Terminology Criteria for Adverse Events (version 4.0) [17]. To assess the correlation on the number of AEs with crizotinib trough concentration, the trough concentrations were divided into a higher trough concentration group (n = 5) and a lower trough concentration group (n = 4) delimited at the median concentration (508.5 ng/mL).

Correlations of crizotinib trough concentration with patient data [height, weight, body mass index (BMI), and body surface area (BSA)] were all determined using the Pearson’s correlation coefficient. The differences in the number of AEs between the patients in the higher and in the lower trough concentration groups were evaluated by the Mann–Whitney *U* test. A *p* value less than 0.05 was considered significant.

### Determination of crizotinib in the plasma of patients

Crizotinib was measured in the plasma samples using a LC-MS/MS method that we developed. One hundred μL of plasma was mixed with an internal standard (IS; erlotinib) solution, and then 200 μL of methanol was added for the deproteinization step. The mobile phase was composed of 0.1% formic acid in a water and acetonitrile mixture (70:30, v/v). The ion transitions for crizotinib and the IS were 451.1/261.0 and 394.1/177.0, respectively. The detection was linear in human plasma at concentrations ranging from 10 to 1000 ng/mL for crizotinib (y = 0.0009 x − 0.0021; *r* = 0.999, *p* < 0.001). The assay accuracy at 5, 10, 100, 200, 500, and 1000 ng/mL ranged from 95.6 to 104.1% (intra-assay) and from 95.9 to 104.1% (inter-assay), respectively. The assay precision at 5 different concentrations ranged from 1.5 to 4.5% (intra-assay) and from 3.2 to 4.4% (inter-assay), respectively.

## Results

### Patient demographic characteristics

Table 1 shows the characteristics of the 9 patients. Patients had been diagnosed with either postoperative recurrence or metastatic NSCLC which were all adenocarcinoma, and all patients had ALK fusion gene-positive NSCLC without epidermal growth factor receptor mutations. Patients #2 and #5 had no past surgical history, and patients #2 and #8 had no previous history of chemotherapy before receiving crizotinib.

**Table 1:** **Patient characteristics**

**Patient**	**Age**	**Sex**	**Height (cm)**	**Weight (kg)**	**BMI** **(kg/m** ^**2**^ **)**	**BSA** **(m** ^**2**^ **)**	**PS**	**Disease stage**	**Previous chemotherapy**
1	41	F	154.2	43.6	18.3	1.38	1	IV	CDDP + DTX, VNR + GEM, PEM
2	40	M	173.5	61.2	20.3	1.73	1	IV	No previous chemotherapy.
3	62	M	175.5	81.3	26.4	1.97	0	IIIA	CBDCA + PTX, CDDP + DTX, Bevacizumab, CPT-11 + AMR + Bevacizumab
4	75	F	146.4	36.8	17.2	1.24	1	IIIA	CBDCA + PEM
5	52	F	158.0	64.2	25.7	1.65	1	IV	CDDP + PEM, PEM
6	57	F	147.5	47.9	22.0	1.39	1	IIIA	CBDCA + PTX, CDDP + VNR
7	57	F	155.2	35.1	14.6	1.26	1	IIA	CBDCA + PTX
8	67	F	146.5	42.4	19.8	1.31	1	IV	No previous chemotherapy
9	54	F	157.8	58.9	23.7	1.59	1	IV	CDDP + PEM, PEM

BMI; body mass index, BSA; body surface area, PS; performance status, CDDP; cisplatin, DTX; docetaxel, VNR; vinorelbine, GEM; gemcitabine, PEM; pemetrexed, CBDCA; carboplatin, PTX; paclitaxel, CPT-11; irinotecan, AMR; amrubicin.

### The crizotinib trough concentration and adverse events

**Figure 1:**
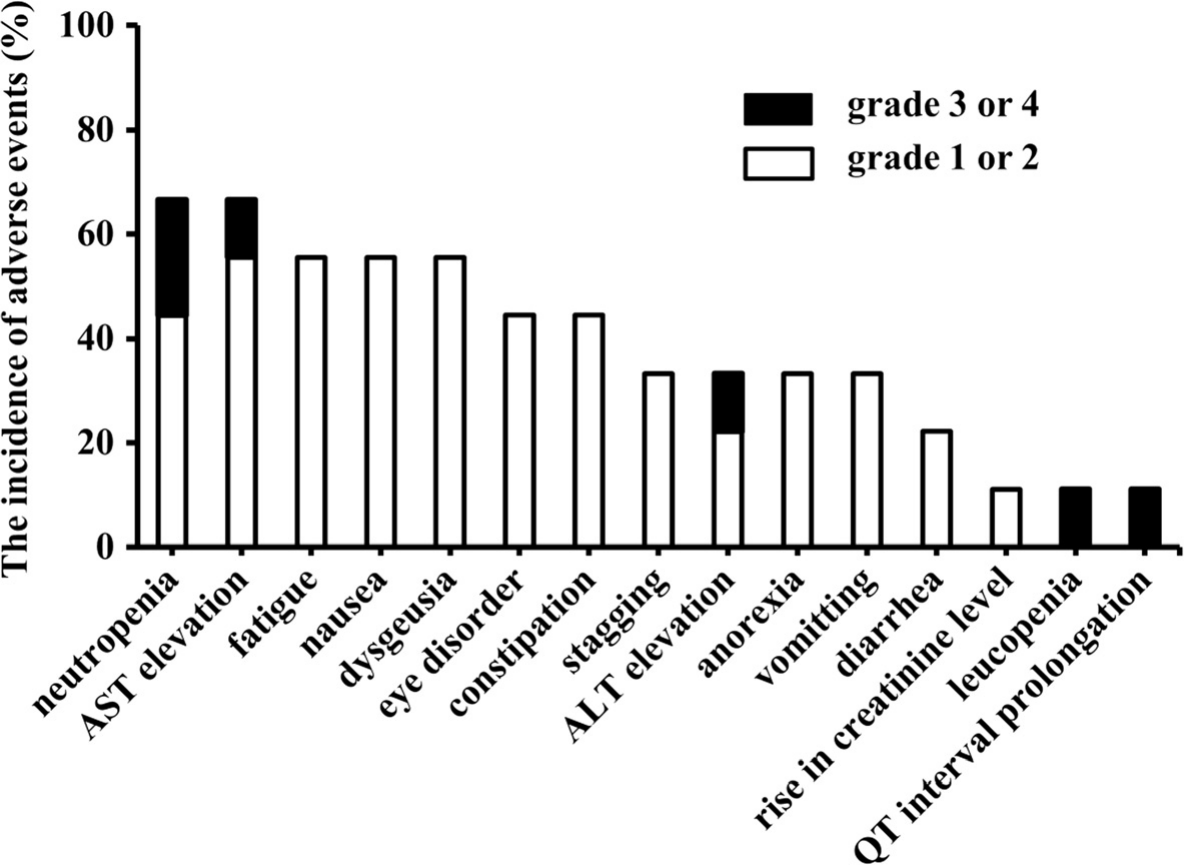
**The incidence of adverse events in patients.** The incidence of AEs was assessed on treatment days 7, 14, 21, and 28. The grades of AEs were assessed using the grading system of Common Terminology Criteria for Adverse Events (version 4.0).

**Figure 2:**
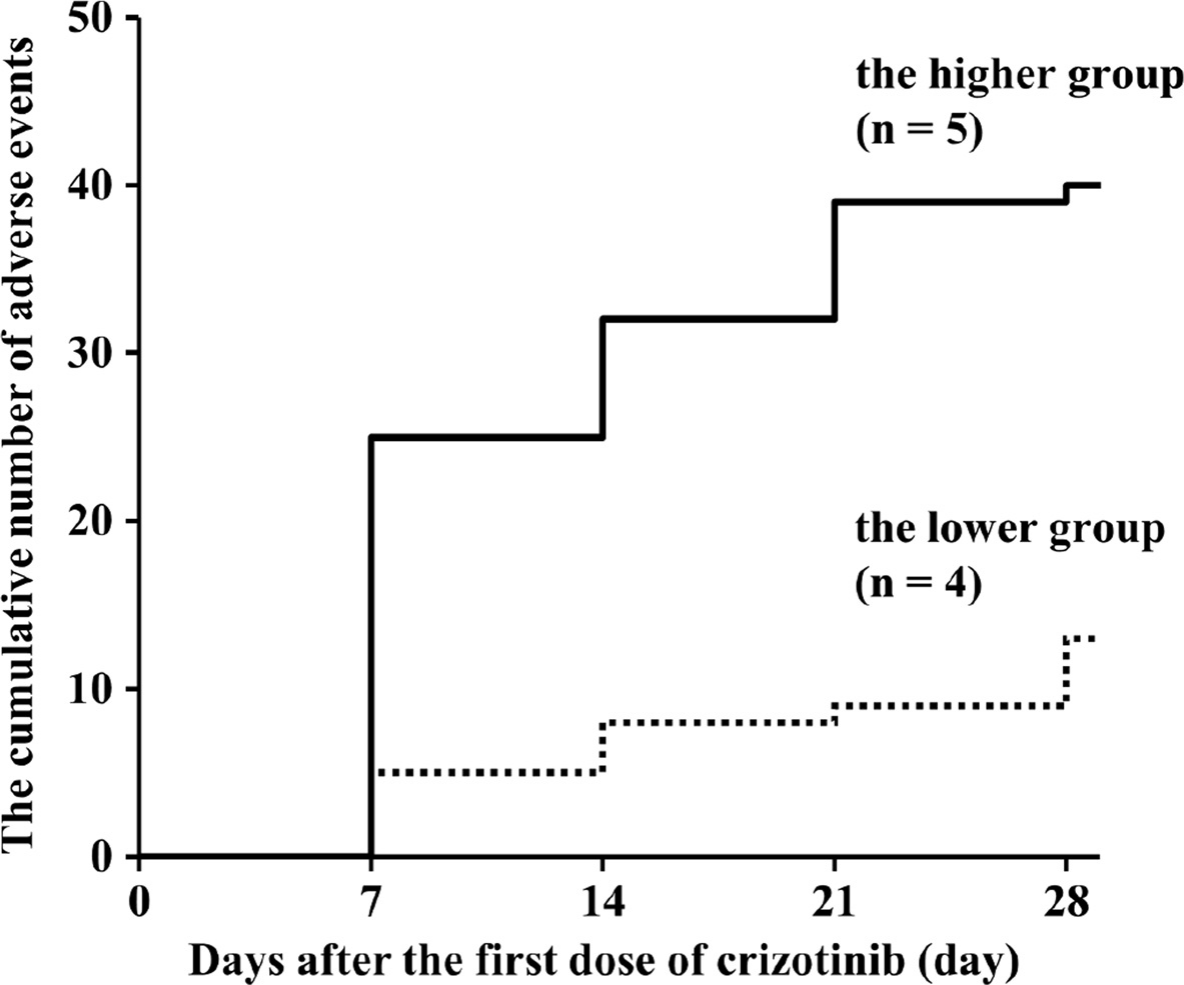
**The cumulative numbers of adverse events in the higher and in the lower crizotinib trough concentration groups.** The crizotinib trough concentration is divided into a higher trough concentration group (n = 5, −−−−−−−) and a lower trough concentration group (n = 4, −------) delimited at the median concentration (508.5 ng/mL). Adverse events were evaluated four times (day 7, 14, 21, and 28).

Nine analytes collected on day 14 were treated as the crizotinib trough concentrations. The median crizotinib trough concentration was 508.5 ng/mL with a range from 243.5 to 847.8 ng/mL. Tumor responses in all of the patients on day 28 were considered as stable disease independent to the crizotinib trough concentration. The AEs observed in the patients on day 7, 14, 21, and 28 are shown in Table 2. The most frequent AEs were neutropenia and AST elevation (Figure 1). The cumulative number of AEs on day 28 in the higher trough concentration group was approximately 3-fold greater than that in the lower trough concentration group (Figure 2). AEs of grade 3 or 4 were observed only in the patients in the higher trough concentration group. However, a significant difference in the number of AEs between the higher and the lower trough concentration groups was not observed.

**Table 2:** **Quantification of crizotinib in the plasma of patients, adjusted doses, and adverse events**

**Patient**	**Daily dose of crizotinib** ^**a**^ **(mg/day)**	**Trough concentration (ng/mL (μM))**	**Tumor response on day 28**	**Crizotinib-induced adverse events** ^**b**^
1	400^a^	589.3 (1.31)	SD	Fatigue, staggering, dysgeusia, neutropenia***, AST elevation
2	500	314.1 (0.70)	SD	Nausea, neutropenia
3	500	370.8 (0.82)	SD	Fatigue, AST and ALT elevations
4	500	825.2 (1.83)	SD	Fatigue, staggering, eye disorder, dysgeusia, nausea, anorexia, AST elevation, constipation
5	500	847.8 (1.88)	SD	Fatigue, eye disorder, dysgeusia, nausea, vomiting, constipation, diarrhea, rise in creatinine levels, leukopenia***, neutropenia****, AST and ALT elevations
6	500	470.8 (1.05)	SD	Fatigue, eye disorder, dysgeusia, constipation, diarrhea, neutropenia, AST elevation
7	500	573.6 (1.27)	SD	Staggering, anorexia, dysgeusia, nausea, constipation, neutropenia, vomiting, AST elevation, QT interval prolongation***
8	500	508.5 (1.13)	SD	Nausea, vomiting, anorexia, neutropenia, AST and ALT elevations***
9	500	243.5 (0.54)	SD	Eye disorder

AST; aspartate aminotransferase, ALT; alanine aminotransferase, SD; stable disease.

^a^Plasma collection was conducted after the dosage had been adjusted due to crizotinib-induced adverse events. The plasma of patients was collected at day 14 before crizotinib was taken in the morning and was analyzed as the trough sample.

^b^All adverse events developed in each patient at days 7, 14, 21 and 28 were listed.

***is grade 3, ****is grade 4, and other adverse events were grade 1 or 2.

## Discussion

Although all of the patients presented with variable crizotinib trough concentrations on day 14, they achieved stable disease according to the assessment of tumor response on day 28. In addition, there was a difference in the frequency of AEs on day 28 between the higher and the lower crizotinib trough concentration groups.

Crizotinib shows promising results in the treatment of ALK fusion gene-positive NSCLC with a recommended dose of 500 mg/day [18]. However, sensitivity to crizotinib may differ among individuals due to many determinants, and may result in developing several different AEs. Based on data from a previous clinical trial of crizotinib, the median trough concentration of crizotinib at a steady-state ranged from 242 to 319 ng/mL [19]. Therefore, some patients in our study might have maintained an elevated crizotinib trough concentration. One crizotinib clinical trial has reported that the Cmax and AUC of crizotinib in Asian patients were 1.57- and 1.50-fold that in non-Asian patients, respectively, and body weight and BSA are thought to be important factors for the pharmacokinetic parameters of crizotinib [10,16,19]. In our study, height, weight, BMI, and BSA with the patients in the higher trough concentration group were likely to be smaller than that with the patients in the lower trough concentration group, however, a significant difference was not observed. Patients in the higher trough concentration group were likely to experience a greater cumulative number of AEs, some of which were grade 3 or 4. These AEs included neutropenia, leucopenia, AST and ALT elevation, and QT interval prolongation. On the other hand, patients in the lower trough concentration group experienced AEs of only grade 1 or 2 during the monitoring period and were more able to tolerate the crizotinib treatment. Therefore, the occurrence and worsening of AEs could be related to the increase in the crizotinib trough concentration. A dose of crizotinib adjusted to the individual patients might be needed for efficient treatment. A limitation of our study was the paucity of patients which was related to the ALK gene rearrangement is observed in 3-7% of NSCLC [3]. Multicenter study would be needed to demonstrate the relationship between the plasma concentration of crizotinib and AEs.

In conclusion, an increasing crizotinib trough concentration might lead to multiple AEs of various grades. The monitoring of crizotinib trough concentration could predict the risk of development of AEs and be a guide for determining the optimal dose of crizotinib.

## Abbreviations

AEs: Adverse events
ALK: Anaplastic lymphoma kinase
ALT: Alanine aminotransferase
AST: Aspartate aminotransferase
AUC: Area under the concentration-time curve
BMI: Body mass index
BSA: Body surface area
Cmax: Maximum drug concentration
c-MET: Hepatocyte growth factor receptor
IS: Internal standard
LC-MS/MS: Liquid chromatography with tandem mass spectrometry
NSCLC: Non-small-cell lung cancer
PS: Performance status
TNM: Tumor, lymph nodes and metastasis
